# Simultaneous Quantification and Visualization of Photosynthetic Pigments in *Lycopersicon esculentum* Mill. under Different Levels of Nitrogen Application with Visible-Near Infrared Hyperspectral Imaging Technology

**DOI:** 10.3390/plants12162956

**Published:** 2023-08-16

**Authors:** Jiangui Zhao, Ning Chen, Tingyu Zhu, Xuerong Zhao, Ming Yuan, Zhiqiang Wang, Guoliang Wang, Zhiwei Li, Huiling Du

**Affiliations:** 1 College of Agricultural Engineering, Shanxi Agricultural University, Jinzhong 030801, China; zhaojiangui1004@126.com (J.Z.); cn15235488116@163.com (N.C.); ztyaiyi@163.com (T.Z.); 18234406740@163.com (X.Z.); ym15735467880@126.com (M.Y.); wzq13753221997@163.com (Z.W.); 2 Institute of Millet Research, Shanxi Agricultural University, Changzhi 046000, China; wangguoliangwz@126.com; 3 College of Information Science and Engineering, Shanxi Agricultural University, Jinzhong 030801, China; 4 Department of Basic Sciences, Shanxi Agricultural University, Jinzhong 030801, China

**Keywords:** hyperspectral imaging, photosynthetic pigments, leaf positions, characteristic variable selection, visualization

## Abstract

Leaf photosynthetic pigments play a crucial role in evaluating nutritional elements and physiological states. In facility agriculture, it is vital to rapidly and accurately obtain the pigment content and distribution of leaves to ensure precise water and fertilizer management. In our research, we utilized chlorophyll a (Chla), chlorophyll b (Chlb), total chlorophylls (Chls) and total carotenoids (Cars) as indicators to study the variations in the leaf positions of *Lycopersicon esculentum* Mill. Under 10 nitrogen concentration applications, a total of 2610 leaves (435 samples) were collected using visible-near infrared hyperspectral imaging (VNIR–HSI). In this study, a “coarse–fine” screening strategy was proposed using competitive adaptive reweighted sampling (CARS) and the iteratively retained informative variable (IRIV) algorithm to extract the characteristic wavelengths. Finally, simultaneous and quantitative models were established using partial least squares regression (PLSR). The CARS–IRIV–PLSR was used to create models to achieve a better prediction effect. The coefficient determination (R^2^), root mean square error (RMSE) and ratio performance deviation (RPD) were predicted to be 0.8240, 1.43 and 2.38 for Chla; 0.8391, 0.53 and 2.49 for Chlb; 0.7899, 2.24 and 2.18 for Chls; and 0.7577, 0.27 and 2.03 for Cars, respectively. The combination of these models with the pseudo-color image allowed for a visual inversion of the content and distribution of the pigment. These findings have important implications for guiding pigment distribution, nutrient diagnosis and fertilization decisions in plant growth management.

## 1. Introduction

Nitrogen is an indispensable element for the growth of green plants as it is involved in the synthesis of photosynthetic pigments, photosynthesis, yield and quality. The administration of the appropriate nitrogen can boost the synthesis of photosynthetic pigments, improving the efficiency of photosynthesis. This process can aid in the synthesis of organic compounds, which ultimately encourage the growth and development of plants [1,2,3]. Plants with low nitrogen levels restrict the production of photosynthetic pigments, resulting in the yellowing of leaves and stunted growth [4,5]. Excessive nitrogen inhibits the absorption of other essential elements, such as potassium and magnesium, leading to a deficiency of these elements [4,5]. This shortage, in turn, has an impact on the physiological function. Furthermore, it reduces the quality of the flowers and fruits, as well as the ability to resist pests and diseases. The growth and development of plants can be affected by varying levels of nitrogen. Notably, there is a positive correlation between the concentration of photosynthetic pigment and the nitrogen status. The determination of the pigment concentration in leaves can serve as an indirect indicator of nitrogen diagnosis. Nitrogen is readily transported in plants, leading to variations in the pigment concentration at different leaf positions [6]. Regarding the detection of pigment, the traditional laboratory chemical analysis remains the predominant method. Although this approach produces accurate and intuitive results, it is also time-consuming, destructive and environmentally unfriendly. Obtaining accurate results for large samples promptly is even more challenging. This hysteresis not only has an impact on agricultural production, but also makes it difficult for agricultural managers to make scientifically sound decisions. Visible-near infrared (VNIR) spectroscopy is a rapid, efficient and non-destructive inspection method that has been used to obtain differences in the pigment concentration in plant leaves [7]. As a result, spectrum analysis has become a prominent research topic for scholars both domestically and abroad in recent years.

The visible-near infrared hyperspectral imager is a non-contact technology that integrates the spectrum and images, making it a powerful tool for remote sensing imaging, environmental monitoring, agricultural production, medical diagnosis and other fields [8,9,10,11,12]. Visible-near infrared hyperspectral imaging (VNIR–HSI) technology provides an effective method for the quantitative and qualitative analysis of crop growth monitoring, quality detection and nutritional diagnosis into agricultural production. The spectral characteristics of various substances are qualitatively analyzed through the segmentation of an HSI. As a result, it is utilized to make assumptions about the characteristics and components of various chemicals in the image to accomplish the classification and identification of substances [13,14,15]. The mathematical inversion model of the image is created by the spectrum data and the statistical analysis of the composition for the material to acquire precise and quantitative information on the substance [16]. Producers can accurately evaluate the growth state, quality features and nutrient level using reflectance spectrum analysis, enabling them to perform precision management of the environment and fertilization. The fundamental components of photosynthesis in green plants are photosynthetic pigments, primarily total chlorophyll (Chls) and total carotenoids (Cars). These molecules can absorb light energy and convert it into chemical energy to promote the process of photosynthesis. Chls, comprising mainly chlorophyll a (Chla) and chlorophyll b (Chlb), is the main light-capturing molecule that absorbs light energy and transfers it to the reaction center to catalyze the process [16]. Meanwhile, Cars plays a supporting part. Different photosynthetic pigments in leaves collaborate to regulate photosynthesis to achieve the best photosynthetic efficiency and growth condition. Thus, the leaf pigment concentration is directly tied to the photosynthetic rate and directly impacts plant growth and development. VNIR–HSI is performed on plant leaves to determine the spatial distribution and spectrum features. The accuracy and quality of the data are improved through various processing techniques such as denoising, calibration, dimensioning and so on [17,18,19,20,21,22]. Vahtmäe et al. [23] established a prediction model for the concentrations of Chla, Chlb, Chls and Cars in various Baltic Sea Macroalgae, demonstrating that VNIR–HSI technology may be effectively applied to the detection of photosynthetic pigment. Wang et al. [24] took Chla, Chlb, Chls and Cars in tea under various nitrogen treatment levels, as the research indicates. Combined with VNIR–HSI technology and the feature selection algorithm to construct prediction models, the correlation coefficients of each pigment prediction were greater than 0.9. This demonstrates that VNIR–HSI can be applied to the rapid and accurate prediction of the pigment concentration. Che et al. [25] analyzed the phenotypes of photosynthetic pigments in *Neopyropia yezoensis* for phycoerythrin (PE), phycocyanin (PC), allophycocyanin (APC) and Chla using VNIR–HSI. Two machine learning techniques, partial least squares regression (PLSR) and support vector machine regression (SVR), were employed in the prediction model after several pre-processing approaches. The quantitative inversion of the photosynthetic pigment concentration through HSI has been the subject of various research works. However, the visual distribution of photosynthetic pigments in leaves has received few investigations. Moreover, crop canopy is the primary focus of the majority of the pertinent studies. Visual distribution can clearly show the distribution of pigments in various leaf positions, as well as the mechanisms of cooperation. Yin et al. [26] suggested that exploring the spatial distribution of crop pigments is a valuable direction for future studies. We can then study the crop physiology and metabolism in depth by discovering the differences in the pigment distribution at different leaf positions. It has vast potential for optimizing resource utilization, improving crop health and advancing precision agriculture practices.

**Figure 1:**
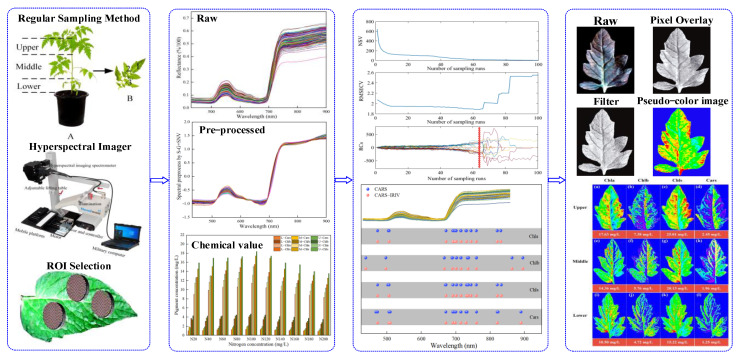
The schematic diagram of this work (**A**,**B**).

This work aimed to use VNIR–HSI technology to simultaneously measure and visualize the distribution of the pigment concentration in *Lycopersicon esculentum* Mill. leaves. More specifically, the research objects were the leaves of *Lycopersicon esculentum* Mill. seedlings cultivated in nutritional solutions with variable nitrogen concentrations at various leaf positions. Figure 1 shows the schematic diagram of this work.

1. The concentration of Chla, Chlb, Chls and Cars were used as the research indexes. The chemical constituents of the leaves were determined using traditional laboratory methods, and the spectrum and image were obtained through VNIR–HSI;
2. The software of batch extraction and processing was compiled for selecting the regions of interest (ROI);
3. A “coarse-fine” characteristic variable screening strategy was proposed for the pre-treated spectral data to establish quantitative models that could simultaneously predict multiple pigments;
4. The distributions and concentrations of pigments at various leaf locations were visualized using prediction models applied to the image. Visual analysis was conducted to determine the appropriate nitrogen concentration for *Lycopersicon esculentum* Mill. cultivation, as well as the distribution of Chla, Chlb, Chls and Cars.

The significance of this study is, on one hand, to use hyperspectral imaging technology combined with a machine learning algorithm to construct pigment predictive models and carry out visual expression. On the other hand, the optimum nitrogen concentration of *Lycopersicon esculentum* Mill. nutrient solution in facility agriculture has been explored to provide a scientific basis for precise fertilizer management in facility agriculture.

## 2. Results

### 2.1. Statistical Analysis of Measured Pigments Concentration of Leaf Positions

**Figure 2:**
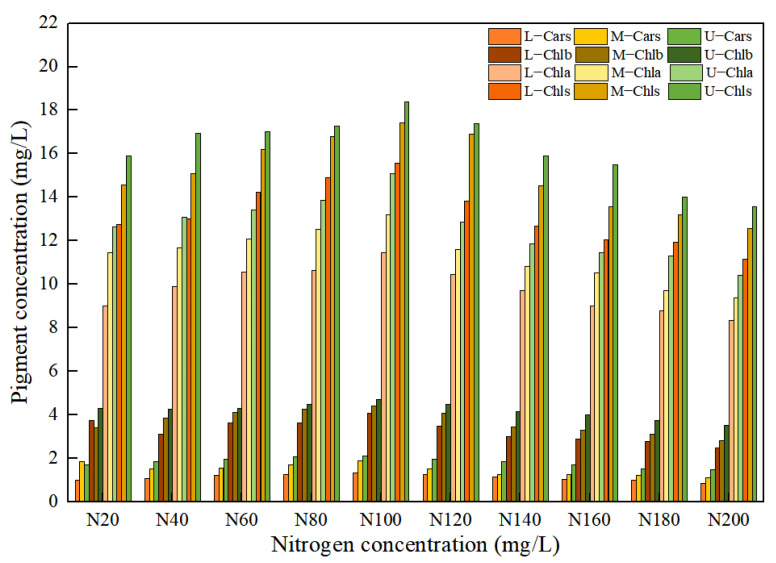
Effect of nitrogen concentration on pigment concentration in *Lycopersicon esculentum* Mill. leaves.

The different nitrogen concentrations in the nutrient solution caused significant differences in the pigment concentration of the leaf positions for *Lycopersicon esculentum Mill*, as shown in Figure 2. The maximum growth rate with the increasing concentration was 7.32%, 11.56%, 7.70% and 10.44% for Chla, Chlb, Chls and Cars, respectively, at nitrogen concentrations below N100 (302.84 mg/L). When the nitrogen concentration was greater than N100, the maximum rate of decline with the increasing concentration reached 11.32%, 12.46%, 11.63% and 8.56%, respectively. As a result, the maximum amount of leaf pigment and the optimal nitrogen concentration for the *Lycopersicon esculentum* Mill. seedlings was 302.84 mg/L in the nutrient solution. Meanwhile, the inhibitory effect of a high concentration was stronger than that of a low concentration. As nitrogen is readily translocated in the plant organism, the concentration reduction rates from Upper to Middle for Chla, Chlb, Chls and Cars were 11.48%, 15.13%, 7.55% and 18.22%, respectively. The reduction rates from Middle to Lower were 15.47%, 12.41%, 14.13% and 22.52%, respectively. Therefore, the distribution pattern of the leaf pigment concentration was Upper > Middle > Lower. This conclusion is the absorption of nutrient elements through the photosynthesis of crops.

### 2.2. Sample Partition and Data Preprocessing

This work aimed to construct simultaneous quantification models for Chla, Chlb, Chls and Cars. In model development, calibration is required to construct the model and perform cross-validation, while prediction is used to evaluate the predictive performance of the model. The SPXY algorithm is used to divide the samples into calibration and prediction sets based on their spectral characteristics and chemical compositions [27,28], ensuring that the distribution of the samples in each data set is roughly balanced in terms of distinctive qualities. All of the samples (*N* = 435) were separated into two datasets, with 326 samples (75% of the total) and 109 samples (25% of the total) constituting the calibration and prediction datasets, respectively. Different spectral pretreatment methods were adopted for different pigment indicators. For different pigment markers, various pretreatment techniques were used. No-pretreatment and pretreatment spectral variables were predicted using PLSR [29,30], and the results are shown in Table S1. The S–G + SNV pretreatment with R_p_^2^ = 0.8064, RMSE_p_ = 1.49 and RPD = 2.27 produced the best results for Chla. The S–G pretreatment with R_p_^2^ = 0.8286, RMSE_p_ = 0.54 and RPD = 2.42 produced the best results for Chlb. The SNV pretreatment with R_p_^2^ = 0.7776, RMSE_p_ = 2.29 and RPD = 2.12 produced the best results for Chls. The SNV pretreatment with R_p_^2^ = 0.7294, RMSE_p_ = 0.29 and RPD = 1.92 produced the best results for Cars. Table 1 displays the statistical reference values for the various pigment indicators in leaves using the best pretreatment strategy. The similarity between the sample mean of the calibration and prediction indicates the rationality of the dataset partitioning.

### 2.3. Results of CARS–PLSR Modeling

The purpose of the CARS algorithm is to eliminate irrelevant variables and reduce the co-linearity between variables. Figures S1–S4 describes the changes in the number of sample variables (NSV), root mean square error of cross-validation (RMSE_cv_) and regression coefficient path (RCs) in the subset as the number of MCS runs increases. As the number of MCS runs increased, the filtered variables declined exponentially at first and then gradually leveled off. The RMSE_cv_ showed a trend of decreasing and then increasing. This indicated that the variables that were removed at the beginning of the variable screening process were not correlated with the components to be measured. Subsequently, the unrelated variables were added to the subset of variables. More effective wavelengths have been retained in the RCs annotation. At this time, the RMSE_cv_ reached the minimum value and the selected variables were the optimal variable set. For Chla, the RMSE_cv_ was at the minimum value when the sampling frequency was 64. The prediction model was created with 16 variables in the subset (R_p_^2^ = 0.8168, RMSE_p_ = 1.45, RPD = 2.34), as shown in Figure S1 and Table S3 and Table 2. For Chlb, when the sampling frequency was 64, the RMSE_cv_ was at the minimum value. The prediction model was created with 16 variables in the subset (R_p_^2^ = 0.8302, RMSE_p_ = 0.54, RPD = 2.43), as shown in Figure S2 and Table S3 and Table 2. For Chls, when the sampling frequency was 61, the RMSE_cv_ was at the minimum value. The prediction model was created with 19 variables in the subset (R_p_^2^ = 0.7869, RMSE_p_ = 2.25, RPD = 2.17), as shown in Figure S3 and Table S3 and Table 2. For Cars, when the sampling frequency was 62, the RMSE_cv_ was at the minimum value. The prediction model was created with 18 variables in the subset (R_p_^2^ = 0.7532, RMSE_p_ = 0.28, RPD = 2.01), as shown in Figure S4 and Table S3 and Table 2.

### 2.4. Results of CARS–IRIV–PLSR Modeling

**Figure 3:**
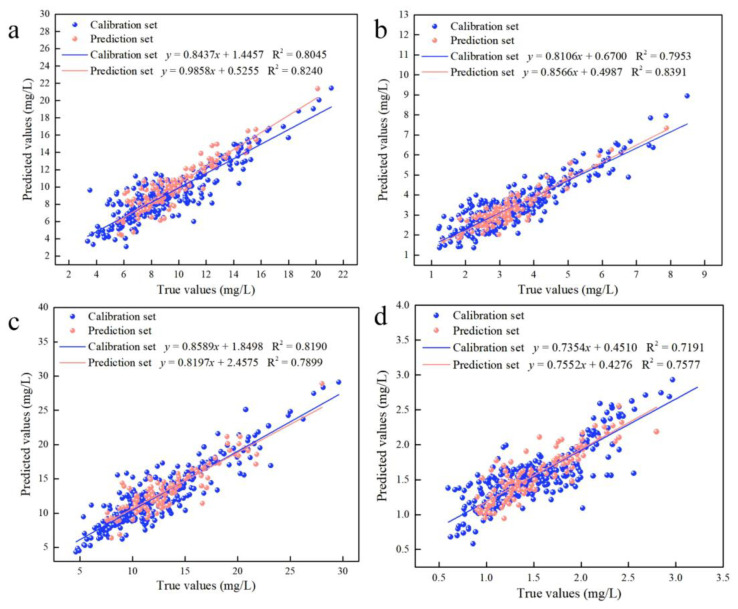
Results of the calibration set and prediction set for the CARS–IRIV–PLSR model of leaf pigment concentration. (**a**) Predicted results of Chla; (**b**) Predicted results of Chlb; (**c**) Predicted results of Chls; (**d**) Predicted results of Cars.

Considering the existence of adjacent variables in the characteristic variables screened by CARS, there may be redundant variables present. The IRIV algorithm establishes a series of sub-models based on a randomly generated subset of sample variables. Variables that appear more frequently in the sub-models have higher weights and are retained during multiple iterations. However, this algorithm requires multiple iterations, resulting in a relatively large computational workload. This work combines the CARS and IRIV algorithms to “exploit strengths and avoid weaknesses”. The IRIV algorithm was used to screen the characteristic variables of the CARS algorithm. Furthermore, the variables selected using the CARS algorithm were determined to be strong informative variables, weak informative variables, uninformative variables and interfering variables. Table S2 describes the classification of the variables screened by the CARS algorithm using the IRIV algorithm. The results showed that only strong and weak variables existed in the variables selected by the CARS algorithm after judging by the IRIV algorithm. The optimal combination of variables was obtained by cross-validating the inverse elimination of the combination of strong and weak information variables using the IRIV algorithm, as shown in Table S3. Table 2 describes the results of the prediction models for Chla, Chlb, Chls and Cars. For Chla, the prediction model was created to achieve the optimal effect with 12 variables using the CARS–IRIV algorithm (R_p_^2^ = 0.8240, RMSE_p_ = 1.43, RPD = 2.38). For Chlb, the prediction model was created to achieve the optimal effect with 10 variables (R_p_^2^ = 0.8391, RMSE_p_ = 0.53, RPD = 2.49). For Chls, the prediction model was created to achieve the optimal effect with 10 variables (R_p_^2^ = 0.7899, RMSE_p_ = 2.24, RPD = 2.18). For Cars, the prediction model was created to achieve the optimal effect with 11 variables (R_p_^2^ = 0.7577, RMSE_p_ = 0.27, RPD = 2.03). The CARS–IRIV–PLSR model was created with the intention of directly expressing the pigment concentration through the prediction performance. The predicted and true values of the calibration set (326 samples) and prediction set (109 samples) are shown in Figure 3.

### 2.5. Visualized Distribution of Leaf Pigments

**Figure 4:**
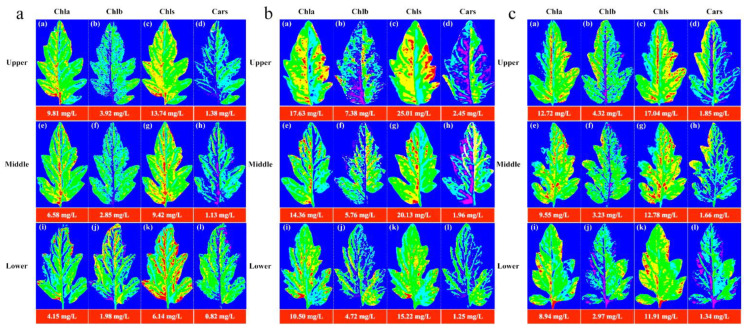
Visualization of pigment concentration and distribution pattern of leaf positions under cultivation with different nitrogen concentrations. (**a**) Visualization of leaf pigments under N60 cultivation; (**b**) Visualization of leaf pigments under N100 cultivation; (**c**) Visualization of leaf pigments under N140 cultivation. (blue represents the minimum value and red represents the maximum value).

The image information of the leaf position of the *Lycopersicon esculentum* Mill. seedlings under different nitrogen concentrations was collected using the HSI system. The leaves from the N60, N100 and N140 cultivations were selected and their contours were extracted using the ENVI software. The characteristic wavelengths were superimposed to calculate the pigment concentration value of each pixel to generate the leaf gray level map using the optimal prediction model of Chla, Chlb, Chls and Cars. The leaf image was inverted and color rendering was applied to the pixels with the concentration value and pseudo-color map to produce a visualization that shows the distribution of the pigment. Figure 4 describes the distribution and concentration of pigments at different leaf locations of the N60, N100 and N140 nitrogen concentrations, respectively. The pigment concentration ranged between 0 and 30 mg/L. The numerical annotation represents the corresponding leaf pigment concentration. The results showed that the concentration of each pigment in the leaves reached the maximum under the N100 concentration treatment, and the pigment concentration was higher under the N60 concentration treatment than under N140. This result further indicates that N100 was the optimum nitrogen concentration for the *Lycopersicon esculentum* Mill. seedlings, while the inhibitory effect was stronger at higher concentrations than at lower ones. The pigment concentration of the leaves showed a pattern of Chls > Chla > Chlb > Cars. The pigment concentration between the leaf locations showed a distribution of Upper > Middle > Lower. This is caused by the absorption of nutrients during the photosynthesis of the crop. The results are in agreement with the chemical measurement. Thus, visualization analysis can clearly elucidate the distribution and concentration of pigments in different leaf positions at different nutrient nitrogen concentrations.

## 3. Discussion

The CARS algorithm adopts EDF to remove the wave points with small regression coefficients, which can enhance the characteristic extraction process. The characteristic wavelengths for Chla, Chlb, Chls and Cars accounted for 2.48% (16/646), 2.48% (16/646), 2.94% (19/646) and 2.79% (18/646) of the full spectrum, respectively. However, there were adjacent wavelengths present in the optimal combination of variables for each pigment correlation. The IRIV algorithm uses the Mann-Whitney U-test to discriminate the degree of correlation of the variables, and the optimal variable set of each pigment has no uninformative or interfering variables. This indicates that the CARS algorithm has certain advantages in characteristic variable extraction. The optimal combination of variables was obtained through the inverse elimination of the set of variables. Finally, the characteristic wavelengths were extracted for Chla, Chlb, Chls and Cars, accounting for 1.86% (12/646), 1.55% (10/646), 1.86% (12/646) and 1.70% (11/646) of the full spectrum, respectively. The characteristic variables extracted were reduced by 0.62%, 0.93%, 1.08% and 1.09%, respectively, compared to those extracted by the CARS algorithm. It is also shown that weak information variables play an important role in the combination, which can effectively improve the predictive performance of the model. From Table 2, it can be concluded that the predicted effect was CARS–IRIV–PLSR > CARS–PLSR > PLSR. This demonstrates the reliability of the “coarse-fine” characteristic extraction strategy of CARS–IRIV proposed in this study. This also reflects the enormous potential of VNIR–HSI technology combined with the CARS–IRIV–PLSR model in the synchronous quantitative detection of pigments.

**Figure 5:**
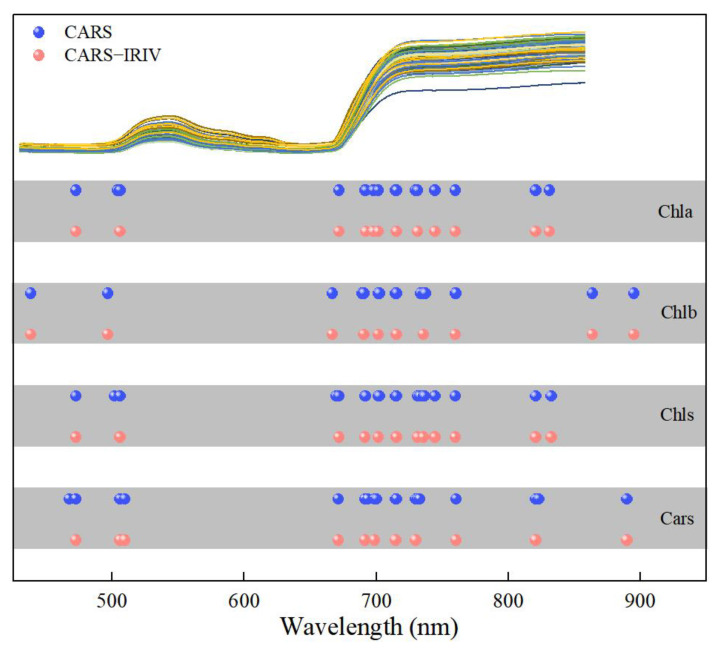
Extraction results of spectral variables for the Chla, Chlb, Chls and Cars in *Lycopersicon esculentum* Mill. leaves.

Figure 5 depicts the distribution of the characteristic variables extracted using the CARS and CARS–IRIV algorithms. The characteristic wavelengths of Chla, Chlb, Chls and Cars were mainly concentrated at 435–510 nm, 660–760 nm and 800–895 nm. And all have 691, 702, 715, 730 and 760 nm selected. In the visible spectral region, Chla, Chlb and Chls mainly absorb blue, purple and red light, with two strong absorption regions at 400–460 nm and 630–680 nm [5,8,31,32]. Cars mainly absorbs blue and purple light, which is a strong absorption region at 400–500 nm [5,10,33,34]. In the near-infrared spectral region, it is mainly caused by the absorption of water or oxygen. Notably, Chla, Chlb, Chls and Cars have overlapping bands selected. The visible region is due to the action of the chromogenic group (C=O, C=C, C≡C) and chromatic group (–OH) [35,36,37,38,39]. In the near-infrared region, it was mainly affected by the O–H bond and O_2_ inside the blade, the double frequency and triple frequency, as well as the combined frequency of the stretching vibration of the C–H group [40].

Chla, Chlb, Chls and Cars are the main photosynthetic pigments in plants. According to Figure 4, the concentration and distribution of pigment in the leaves could be obtained. Chla decreased gradually from the bottom of the leaf to the top and from the vein to the edge of the leaf. This is because Chla is mainly present in the chloroplast thylakoid membrane, while the nutrients are transported to the leaves through the leaf veins [19,41]. Therefore, the Chla concentration was higher in the bottom and vein parts of the leaves. In contrast, the top and leaf edge parts of the leaves had a relatively low nutrient supply, resulting in a relatively low Chla concentration. Chlb is mainly distributed in the photosynthetic membrane of chloroplasts [42]. The distribution in the leaves is heterogeneous, with higher levels around the leaves and in the veins and lower levels in the center. Chls is mainly composed of Chla and Chlb. It is mainly distributed in the chloroplasts, but also in other cytoplasm of the leaves. The concentration of Chlb is much lower than that of Chla, accounting for only 10–15% of the Chls [43], resulting in a distribution pattern of Chls that is roughly consistent with that of Chla. Cars is mainly distributed in the epidermal and subepidermal cells of leaves. *Lycopersicon esculentum* Mill. seedlings are in the flourishing stage of development and photosynthesis promotes the active and high concentration of Chls in leaves. In this process, Cars protects the leaves from UV and free radical damage. Therefore, it is present in high levels in the epidermal and subepidermal cells of the leaves. However, Cars mainly acts on the leaf color and their concentration is low, resulting in Cars being covered by Chls. It is similar to the distribution of Chlb in leaves. The differences in the distribution of pigments in the leaf positions are due to differences in the nutrient transport and light exposure, which are consistent with the physiological characteristics of the plants.

## 4. Materials and Methods

### 4.1. Experimental Design and Sample Collection

The experiment was carried out in the scientific greenhouse of the College of Agricultural Engineering, Shanxi Agricultural University (37°25′ N, 112°34′ E), from 12 November 2021 to 13 December 2021 and 25 September 2022 to 18 October 2022, respectively. The seedling of *Lycopersicum esculentum Mill*. *cv. Provence* with “4 leaves and 1 core” was purchased from the nursery company. A total of 145 plants were transplanted into a nutritive bowl with coconut bran as the substrate. The nutrient solution adopted the *Lycopersicum esculentum Mill* formula self-prepared water-soluble fertilizer of the Holland Greenhouse Horticulture Research Institute, with 10 nitrogen gradients (denote as N20, N40, N60, N80, N100, N120, N140, N160, N180, N200). Ca^2+^ was supplemented with calcium fertilizer (Ca^2+^ ≥ 94%, Green-Micro Power Crop Nutrition Co., Northamptonshire, UK) to keep the concentration constant. The nitrogen concentration was regulated using urea, and Table S4 shows its precise addition, EC and pH. The leaves were sampled at the “transplant-bloom” (seedling) stage. At this time, 10–11 branches extended from the main stem. As shown in Figure 1A, the leaves were arranged in descending order, wherein the 9–7 branch blades were classified as the upper leaf location (Upper), the 6–4 branch blades were the middle leaf location (Middle) and the 3–1 branch blades were the lower leaf location (Lower). *Lycopersicum esculentum Mill* is a “single-branch and leafiness” plant. Therefore, sample collection on each branch is referred to in Figure 1B. The leaves with uniform size and spreading were picked based on the sampling rule, with 6 leaves (1 sample) chosen from each leaf location for a total of 2610 leaves (435 samples). The samples were stored in sealed bags with sequential numbers and kept in a dry ice-filled incubator.

### 4.2. Hyperspectral Image Acquisition

Leaf images were collected using the VNIR–HSI (Headwall Photonics, Starter Kit, Bolton, MA, USA) scanning platform. This system captures 856 spectral bands from 380 to 1000 nm with a spectral resolution of approximately 0.727 nm. A spectral range with a total of 646 bands, ranging between 430 and 900 nm, is chosen due to the significant reflectivity error near the measuring range. The movement speed, push-broom stroke and distance between the lens and leaf for the system were set to 2.721 mm/s, 100 mm and 28 cm, respectively, in order to obtain clear and undistorted images. First, wash the dust and impurities off the leaf surface with deionized water. Second, blot the surface moisture with filter paper. Finally, lay the leaves flat on the stage to obtain hyperspectral images. To reduce the image interference generated by the system light source and dark current, the hyperspectral image is corrected for black and white according to the following equation:

(1) $$R=\frac{R_{0}-R_{b}}{R_{w}-R_{b}}$$

where *R* is the corrected hyperspectral image; *R*_0_ is the original hyperspectral image; *R_w_* is the white background image with the standard white calibration plate (>99.9% reflectance); *R_b_* is the dark background image with the lens cap closed (<0% reflectance).

### 4.3. Chemical Measurement of Pigment Concentration

After collecting spectral images from the samples, the concentration of Chla, Chlb, Chls and Cars was measured using an ultraviolet spectrophotometer (Jingke Shangfen, Shanghai, China). After removing the veins, each sample was sliced into pieces of about 2 × 2 mm, mixed evenly, weighed at 0.2 g and deposited in a test tube. It was extracted with 96% ethyl alcohol in the darkness for 24 h until the pieces turned white. The absorbance of the prepared pigment extracts was measured at wavelengths of 665, 649 and 470 nm, respectively [37]. Each sample was repeated 3 times, and the pigment concentration was calculated according to the following formula:

(2) $$Chla=13.95\times A_{665}-6.88\times A_{649}$$

(3) $$\mathrm{Chlb}=24.96\times A_{649}-7.32\times A_{665}$$

(4) Chls=Chla+Chlb

(5) $$\mathrm{Cars}=\left(1000\times A_{470}-2.05\times \mathrm{Chla}-114.8\times \mathrm{Chlb}\right)/245$$

### 4.4. Selection of ROI

On one hand, there are veins of different sizes distributed in the leaves, especially the largest midrib in the center. VNIR–HSI, on the other hand, has a high resolution and lots of pixels. Choosing the ROI for the leaves was challenging due to these factors. The SpectralView software (Headwall Photonics, Bolton, MA, USA) was used for secondary development with Visual Basic (Version 6.0, Microsoft, Redmond, WA, USA), which produced the software for batch extraction and processing of hyperspectral data. The core module of the program was made up of a pixel-generating module and a batch-processing module. The elliptical model was used to determine the coordinates of the center of the ROI, the length of the X/Y semi-axis (*a*, *b*) and the distance between the X/Y axes (Δ*x*, Δ*y* were both set to 1). Following the principle of “from left to right, from top to bottom” in the target image, pixels within the ROI were collected sequentially according to Formula (6) to generate the ROI coordinate matrix. The SpectralView software was used to import images and actively extract the reflectivity information based on the coordinate matrix. Depending on the requirements, the batch processing module could produce numerical calculation results such as mean, mean difference and variance. The leaf is mostly composed of the leaf tip, leaf base, leaf margin and other components. To extract as many of these parts as possible, the leaf was divided into three areas to extract a total of 32,000 pixels. The sample average spectral (Formula (7)) was adopted as the basic dataset for subsequent processing.

(6) $$\frac{x_{i}^{2}}{a^{2}}+\frac{y_{i}^{2}}{b^{2}}\leq 1$$

(7) $$A_{i}=\frac{1}{n}(A_{1}+A_{2}+A_{3}+\ldots +A_{n})=\frac{1}{n}\sum_{i=1}^{n}A_{i,k}$$

### 4.5. Spectrum Pretreatment and Model Calibration

**Figure 6:**
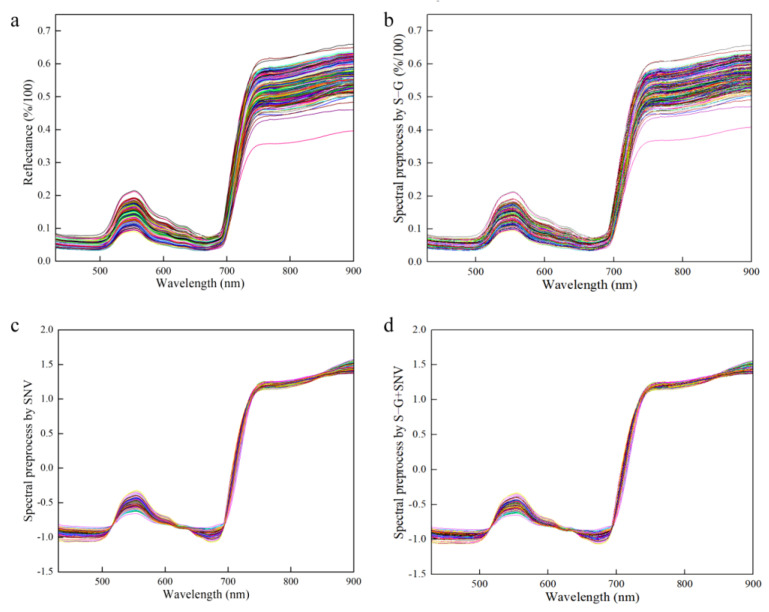
Preprocessed of spectrum data. (**a**) Raw spectrum for all the samples; (**b**) S–G preprocessed spectrum for all the samples; (**c**) SNV preprocessed spectrum for all the samples; (**d**) S–G + SNV preprocessed spectrum for all the samples.

Spectral data contain not only the informative concentration, but also a significant amount of redundant and interfering information. Preprocessing, as a crucial step in data refinement, is utilized to adjust the variability of each measured variable and its relationship to better align with the objectives of the data analysis. The selection and combination of preprocessing methods are dependent on the unique characteristics of the data and the goals of the analysis process. Spectrum stability is always influenced by leaf gloss, leaf reflection, background interference and baseline drift during the scanning process. The Savitzky–Golay smoothing filter (S–G) is capable of mitigating the impact of interfering factors, such as the signal-to-noise ratio and dark current, thereby enhancing the accuracy and reliability of the data analysis [44,45]. The standard normal variable (SNV) is primarily employed to eliminate the effects of particle size, surface scattering and light path variation on diffuse reflection spectra [18,46]. Therefore, the effective spectrum was preprocessed using the S–G, SNV and S–G + SNV approaches, which reduced interference before modeling and effectively improved the prediction accuracy of the model, as shown in Figure 6.

While VNIR–HSI can effectively provide simultaneous data, the numerous variables also result in curses of dimensionality, which reduces the load and predictive capacity of the model. In this study, competitive adaptive reweighted sampling (CARS) and an iterative retained information variable (IRIV) were used to reduce the dimension of variables. This is beneficial to the interpretability of the variables and improves the accuracy of the prediction models.

The CARS algorithm selects the optimal combination of effective variables in the spectrum by imitating the principle of “survival of the fittest” in Darwinian evolution [19,20]. For the spectrum array of *m* × *p* dimensions (*m* represents the number of samples, and *p* represents the number of variables), CARS selected the effective wavelengths through the following steps:

1. Based on Monte Carlo sampling (MCS), a PLSR model is established by randomly selecting 80% of the calibration set of samples to obtain the regression coefficients |*K_i_*| (*i* = 1, 2, ⋯, *p*) for the *i*-th wavelength;
2. The exponentially decreasing function (EDF) is applied to eliminate the wavelength with smaller |*K_i_*|, and the retention rate of the variable is *r_j_* = *a*e^−*bj*^ (*j* = 1, 2, ⋯, *N*). Among them, *j* represents the *j*-th MCS; *N* represents the number of MCS; *a* and *b* are constants, calculated by *r*_1_ = 1 and *r_N_* = 2/*p*, the formula are as follows:
  (8) $$a=(p/2{)}^{1/(N-1)}$$
  (9) b=ln(p/2)/(N−1)
3. The variables are further filtered based on the adaptive reweighted sampling (ARS) technique. The variables were filtered by evaluating the weights $w_{i}=\left|K_{i}\right|/\sum_{i=1}^{p}\left|K_{i}\right|$ (*i* = 1, 2, ⋯, *p*);
4. Repeat the above steps until the number of MCS reaches a predetermined value of *N*;
5. The 5-fold root mean square error of cross-validation (RMSE_cv_) is used as the evaluation criterion. The values of the subset of variables obtained from each MCS are compared, and the subset of variables corresponding to the minimum RMSE_cv_ is selected as the optimal variable.

The iterative retaining information variable (IRIV) algorithm uses random combinations and interactions of variables to extract characteristic variables based on a binary matrix rearrangement filter [21,22]. IRIV takes the following steps to select the effective wavelengths.

1. The spectrum bands randomly generate an *m* × *p* matrix A containing only 0 and 1 (0 and 1 indicate whether the corresponding variables are involved in performing the modeling), with the same number of 0 and 1. The PLSR model is established in each row of matrix A. The RMSE_cv_ obtained from the 5-fold cross-validation is used as the evaluation criterion. This obtains an *m* × 1 vector denoted as RMSE_cv0_. Replace the 1 with 0 and the 0 with 1 in the *i*-th (*i* = 1, 2, ⋯, *p*) column of the A to obtain the matrix B. Similarly, a PLSR model is established in each row of the B to obtain an *m* × 1 vector denoted as RMSE_cv*i*_;
2. Define Φ_0_ and Φ*_i_* to assess the importance of each variable with the following equations. The difference between the mean values of Φ_0_ and Φ*_i_* is denoted as *DM_i_*. If *DM_i_* < 0, it is a strong or weak information variable; if *DM_i_* > 0, it is an uninformative or interfering variable. A Mann-Whitney U-test is performed by defining *p* = 0.05 as the threshold. Finally, the variables are classified as strong information, weak information, uninformative and interfering information:
  (10) $$\Phi_{0K}=\left\{\begin{array}{cc} k^{th}RMSECV_{0} & if\;A_{ki}=1\\ k^{th}RMSECV_{i} & if\;B_{ki}=1 \end{array}\right.\begin{array}{cc} ; & \Phi_{iK}=\left\{\begin{array}{cc} k^{th}RMSECV_{0} & if\;A_{ki}=0\\ k^{th}RMSECV_{i} & if\;B_{ki}=0 \end{array}\right. \end{array};$$
3. In each iteration, strong and weak information variables are retained, and uninformative and interfering information variables are eliminated. Return to step 1. for the next iteration until only strong and weak information is left in the set of variables;
4. Backward elimination is performed for *t* retained variables. First, a PLSR model is established for the *t* variables to obtain RMSE_cvt_. Then, a PLSR model is established for the *t* − 1 variables by eliminating the *j*-th (*j* = 1, 2, ⋯, *t*) variable to obtain RMSE_cv*j*_. If RMSE_cv*j*_ is less than RMSE_cv*t*_, the *j*-th variable is eliminated; otherwise, it is retained. Loop this process, and the remaining variables are the final selected characteristic variables.

In this work, the CARA−IRIV algorithm was proposed by combining the advantages of the rapid iteration rate of CARS and the selection of strong and weak information variables by IRIV. It was used to screen pigment-related variables in leaves, thereby reducing the redundancy of the explainable variables and improving the model performance. Combined with PLSR, the prediction models of CARS–PLSR and CARS–IRIV–PLSR were established, respectively. At the same time, 50 runs were performed on CARS–PLSR and CARS–IRIV–PLSR. The predictive ability of the model was evaluated through the coefficient determination of calibration (R_c_^2^) and prediction (R_p_^2^), root mean square error of calibration (RMSE_c_) and prediction (RMSE_p_) and ratio performance deviation (RPD). When RPD > 2, it indicates that the model has a good prediction of the indicator. When 1.4 < RPD < 2, it indicates that the model has some ability to predict the indicator. When RPD < 1.4, it indicates that the model is unable to predict the indicator [47]. The MATLAB software (Ver. 2018a, MathWorks, Natick, MA, USA) was used to process and analyze the data.

### 4.6. Visualization of Leaf Pigment

Hyperspectral imaging technology perfectly combines spectrum and image information, with each pixel on the image containing a spectrum curve. In addition to detecting the sample index, the detection index can be quantitatively inverted to the sample image combined with the optimal prediction model parameters to realize the visual expression of the index to be measured.

The ENVI software (Ver. 5.1, Harris Geospatial, Broomfield, CO, USA) was used to process the corresponding wavelength multiplied by the weight coefficient to obtain the assignment value of each pixel, i.e., the leaf pigment concentration value. At the same time, the enhanced Lee filter was used to reduce the speckle noise. The filter size, damping coefficient, homogeneity zone and heterogeneous zone were set to 3 × 3, 1, 0.52 and 1.73, respectively. Lastly, the pseudo-color map combined with the assignment size inverting the pixel points on the leaf image produces a visual representation of the concentration distribution.

## 5. Conclusions

In this study, the Chla, Chlb, Chls and Cars of the leaves of *Lycopersicon esculentum* Mill. seedlings were studied under different nitrogen concentration cultivations. VNIR–HSI technology combined with a machine learning algorithm was used to model the prediction of the pigment concentration. Thus, it is convenient to realize the visualization of pigment expression. The nitrogen concentration of the nutrient solution was 302.84 mg/L, and the pigment concentration of the leaves was the largest. The distribution pattern of the pigment concentration in the leaf positions was Upper > Middle > Lower. Meanwhile, the inhibitory effect of a high concentration was stronger than that of a low concentration, which could provide data support for the quantitative management of the nitrogen concentration of water and fertilizer in facility agriculture. The “coarse–fine” characteristic variable extraction strategy of CARS–IRIV is proposed, which can effectively reduce adjacent bands and retain the effective information as much as possible. The PLSR was used to establish the prediction model for each pigment index to achieve better results. The model prediction accuracy was improved after characteristic wavelengths screening. Combining the CARS–IRIV–PLSR model with hyperspectral imaging technology effectively visualizes the expression of leaf pigments. The concentration of pigments and their distribution in leaves can be visualized, which helps to non-destructively monitor the growth condition, pigment concentration and nutrient diagnosis of plants in facility agriculture. For diagnosing plant responses to nutritional stress, HSI is a potential method. The following aspects may be considered for future development:

- Plants may experience various challenges in addition to nitrogen stress, such as salt stress and water stress. In the future, HSI technology can be integrated with other stress monitoring technologies to realize multi-stress joint diagnosis and to better comprehend the growing stage of plants;
- Combining the hyperspectrum, Raman spectrum and fluorescence spectrum to obtain multi-source data allows for the qualitative and quantitative analysis of the stress mechanism;
- HSI superpixel segmentation mostly targets images with a single scale and one mode. The superpixel segmentation of multi-scale and multi-modal pictures will receive more attention in future studies. Super-pixel segmentation uses end-to-end learning techniques to apply deep learning to enhance the accuracy and efficiency as it adapts to more complex image scenarios;
- HSI technique generates a significant amount of complicated data, necessitating the use of effective data processing and analysis algorithms. Future research can concentrate on streamlining data processing techniques to raise the precision and effectiveness of stress diagnosis;
- The primary focus of the current HSI technology is static images of plants; however, future advancements may provide dynamic monitoring to enable the continuous observation of the plant stress response at various periods and spatial scales;
- The primary requirement of the current HSI technology is massive imaging apparatus, which restricts its applicability in real-world field applications. Future research might concentrate on creating more lightweight imaging equipment that is easier to utilize in various settings.

In general, crop stress diagnosis offers a wide range of development opportunities for HSI technologies. As a result, agricultural output and environmental preservation will be supported while also helping to better understand and regulate how plants react to various pressures.

## Figures and Tables

**Table 1:** Statistics of pigments concentration in leaves.

Pigments	Subsets	NS ^a^	Range (mg/L)	Mean (mg/L)	SD ^b^ (mg/L)
Chla	Calibration set	326	3.37–21.13	9.22	3.05
	Prediction set	109	5.56–20.14	9.61	2.52
Chlb	Calibration set	326	1.22–8.49	3.29	1.23
	Prediction set	109	1.80–7.89	3.45	1.04
Chls	Calibration set	326	4.61–29.62	12.44	4.19
	Prediction set	109	7.52–28.02	13.26	5.07
Cars	Calibration set	326	0.6–3.23	1.48	0.48
	Prediction set	109	0.9–2.8	1.50	0.42

^a^ NS: number of samples; ^b^ SD: standard deviation.

**Table 2:** Results of prediction models for different pigments.

Pigments	Models	Calibration Set		Prediction Set		RPD
		R_c_^2^	RMSE_c_	R_p_^2^	RMSE_p_	
Chla	PLS	0.7877	1.88	0.8064	1.49	2.27
	CARS–PLS	0.8040	1.81	0.8168	1.45	2.34
	CARS–IRIV–PLS	0.8045	1.81	0.8240	1.43	2.38
Chlb	PLS	0.7790	0.79	0.8286	0.54	2.42
	CARS–PLS	0.7899	0.77	0.8302	0.54	2.43
	CARS–IRIV–PLS	0.7953	0.74	0.8391	0.53	2.49
Chls	PLS	0.7964	2.53	0.7776	2.25	2.12
	CARS–PLS	0.8185	2.41	0.7869	2.25	2.17
	CARS–IRIV–PLS	0.8190	2.40	0.7899	2.24	2.18
Cars	PLS	0.6768	0.35	0.7294	0.29	1.92
	CARS–PLS	0.7170	0.33	0.7532	0.28	2.01
	CARS–IRIV–PLS	0.7191	0.33	0.7577	0.27	2.03

## Data Availability

Available upon request from the corresponding author.

## Supplementary Materials

The following supporting information can be downloaded at: https://www.mdpi.com/article/10.3390/plants12162956/s1, Table S1: Prediction results of pigments using different pre-processing methods; Table S2: Classification of variables screened by the CARS algorithm using the IRIV algorithm; Table S3. The key wavelengths screened by CARS and CARS–IRIV algorithms for Chla, Chlb, Chls and Cars; Table S4: Fertilizer (mg/L), EC and pH with different nitrogen concentrations; Figure S1: The changes in sample variables (NSV), RMSEcv and regression coefficient paths (RCs) in a subset of CARS arithmetic for Chla; Figure S2: The changes in sample variables (NSV), RMSEcv and regression coefficient paths (RCs) in a subset of CARS arithmetic for Chlb; Figure S3: The changes in sample variables (NSV), RMSEcv and regression coefficient paths (RCs) in a subset of CARS arithmetic for Chls; Figure S4: The changes in sample variables (NSV), RMSEcv and regression coefficient paths (RCs) in a subset of CARS arithmetic for Cars.
