# Supplementary material for: Simultaneous Quantification and Visualization of Photosynthetic Pigments in Lycopersicon esculentum Mill. under Different Levels of Nitrogen Application with Visible-Near Infrared Hyperspectral Imaging Technology
Source: Plants (Basel). 2023 Aug 16;12(16):2956. doi: 10.3390/plants12162956 (PMC10459730; doi:10.3390/plants12162956)
Supplement: Supplementary file 1 [file plants-12-02956-s001.zip › plants-2505452-supplementary.pdf]

plants MDPI

Table S1. Prediction results of pigments using different pre-processing methods.

Pigments	Pretreatment	Calibration set		Prediction set		RPD
		R_c^2	$RMSE_c$	R_p^2	$RMSE_p$	
Chla	Raw	0.7632	1.97	0.7976	1.53	2.22
	S–G	0.7645	1.97	0.7781	1.55	2.12
	SNV	0.8002	1.83	0.7171	1.75	1.88
	S–G+SNV	0.7877	1.88	0.8064	1.49	2.27
Chlb	Raw	0.7863	0.78	0.7831	0.56	2.15
	S–G	0.7790	0.79	0.8286	0.54	2.42
	SNV	0.8036	0.73	0.8223	0.59	2.37
	S–G+SNV	0.7944	0.75	0.8156	0.60	2.33
Chls	Raw	0.7863	2.60	0.7369	2.46	1.95
	S–G	0.7772	2.67	0.7646	2.29	2.06
	SNV	0.7964	2.53	0.7776	2.25	2.12
	S–G+SNV	0.7982	2.53	0.7677	2.29	2.07
Cars	Raw	0.6827	0.35	0.6912	0.29	1.80
	S–G	0.6710	0.36	0.6828	0.29	1.78
	SNV	0.6768	0.35	0.7294	0.29	1.92
	S–G+SNV	0.6689	0.36	0.7213	0.28	1.89

Table S2. Classification of variables screened by the CARS algorithm using the IRIV algorithm.

Pigments	Variable classification	Spectral variables (nm)
Chla	Strong information	473, 504, 672, 698, 702
	Weak information	506, 691, 692, 715, 716, 730, 731, 745, 760, 821, 831
Chlb	Strong information	439, 497, 667, 689, 691, 702, 703, 715, 716
	Weak information	734, 736, 737, 760, 761, 864, 895
Chls	Strong information	473, 502, 670, 692, 702, 703, 745
	Weak information	506, 672, 691, 715, 716, 731, 733, 736, 737, 760, 821, 832
Cars	Strong information	468, 473, 506, 761, 890
	Weak information	510, 671, 691, 694, 699, 700, 715, 716, 730, 731, 733, 821, 823

Table S3. The key wavelengths screened by CARS and CARS–IRIV algorithms for Chla, Chlb, Chll and Caro.

Pigments	VSM[a]	NV[b]	Key spectral variables (nm)
Chla	CARS	16	473, 504, 506, 672, 691, 692, 698, 702, 715, 716, 730, 731, 745, 760, 821, 831
	CARS–IRIV	12	473, 506, 672, 692, 698, 702, 715, 731, 745, 760, 821, 831
Chlb	CARS	16	439, 497, 667, 689, 691,702, 703, 715, 716, 734, 736, 737, 760, 761, 864, 895
	CARS–IRIV	10	439, 497, 667, 691, 702, 715, 736, 760, 864, 895
Chls	CARS	19	473, 502, 506, 670, 672, 691, 692, 702, 703, 715, 716, 731, 733, 736, 737, 745, 760, 821, 832
	CARS–IRIV	12	473, 506, 672, 691, 702, 715, 731, 736, 745, 760, 821, 832
Cars	CARS	18	468, 473, 506, 510, 671, 691, 694, 699, 700, 715, 716, 730, 731, 733, 761,821, 823, 890
	CARS–IRIV	11	473, 506, 510, 671, 691, 699, 715, 730, 761, 821, 890

[a]VSM: variables selection methods; [b]NV: number of variables.

Table S4. Fertilizer (mg/L), EC and pH with different nitrogen concentrations.

Fertilizer	N20	N40	N60	N80	N100	N120	N140	N160	N180	N200
Calcium nitrate	0	307.48	605.68	913.15	1216	1216	1216	1216	1216	1216
Calcium fertilizer	491.57	367.27	246.72	122.43	0	0	0	0	0	0
Urea	0	0	0	0	0	131.67	262.34	395.01	526.68	658.35
Calcium ammonium nitrate	42.1	42.1	42.1	42.1	42.1	42.1	42.1	42.1	42.1	42.1
Potassium nitrate	395	395	395	395	395	395	395	395	395	395
Potassium phosphate monobasic	208	208	208	208	208	208	208	208	208	208
Potassium sulphate	393	393	393	393	393	393	393	393	393	393
Magnesium sulphate	466	466	466	466	466	466	466	466	466	466
Nitrogen concentration	59.64	121.14	181.7	242.27	302.84	363.41	423.98	484.54	545.54	605.68
EC	2.49	2.42	2.40	2.30	2.29	2.33	2.43	2.50	2.53	2.56
pH	6.88	6.84	6.80	6.95	6.99	7.05	7.00	6.95	6.96	6.98

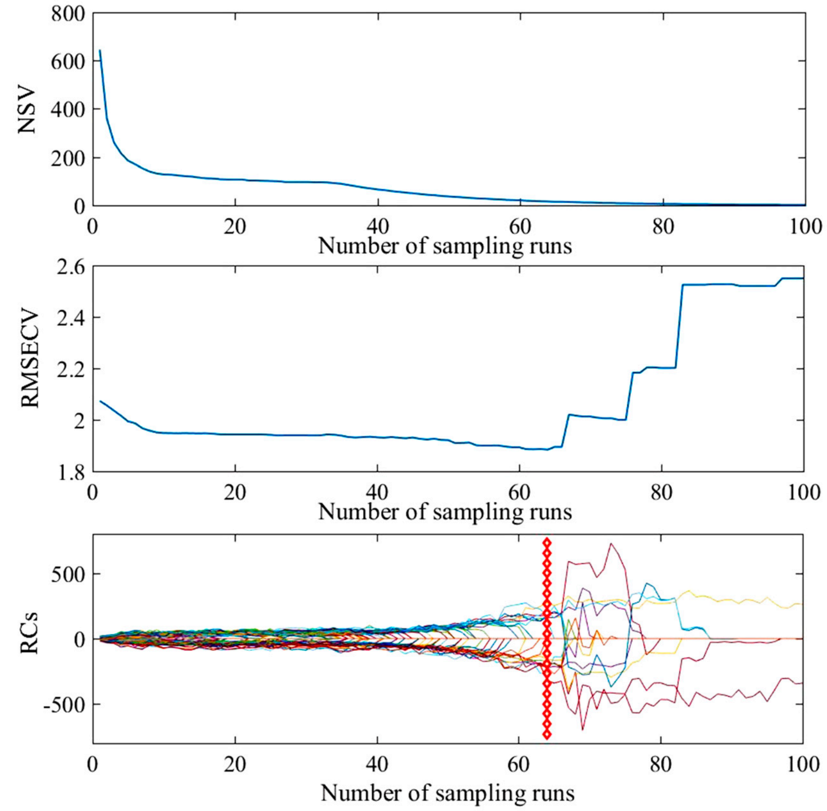

Figure S1. The changes in sample variables (NSV), $RMSE_{cv}$ and regression coefficient paths (RCs) in a subset of CARS arithmetic for Chla.

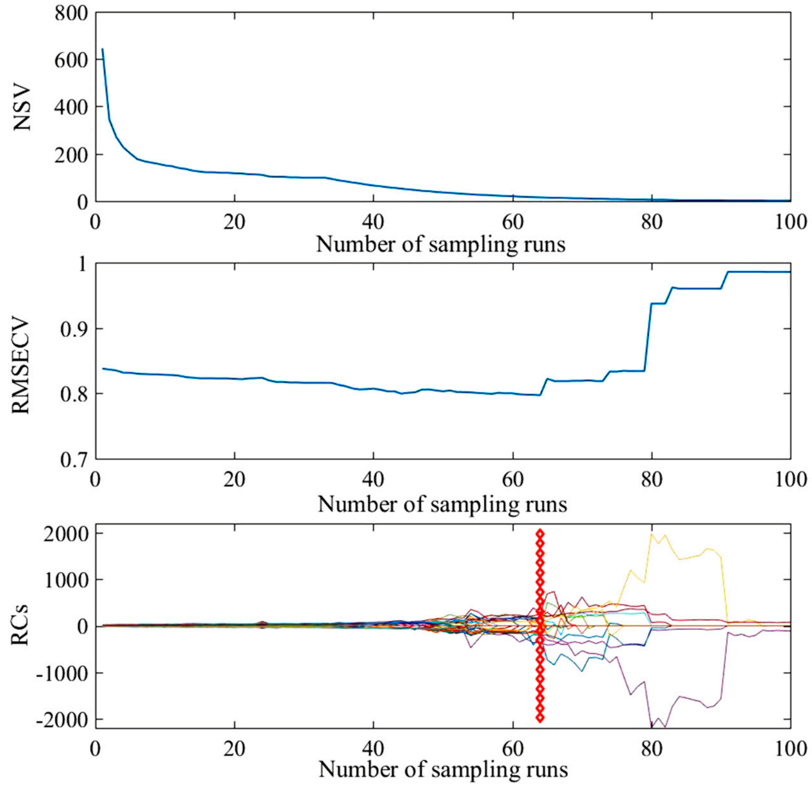

Figure S2. The changes in sample variables (NSV), $RMSE_{cv}$ and regression coefficient paths (RCs) in a subset of CARS arithmetic for Chlb.

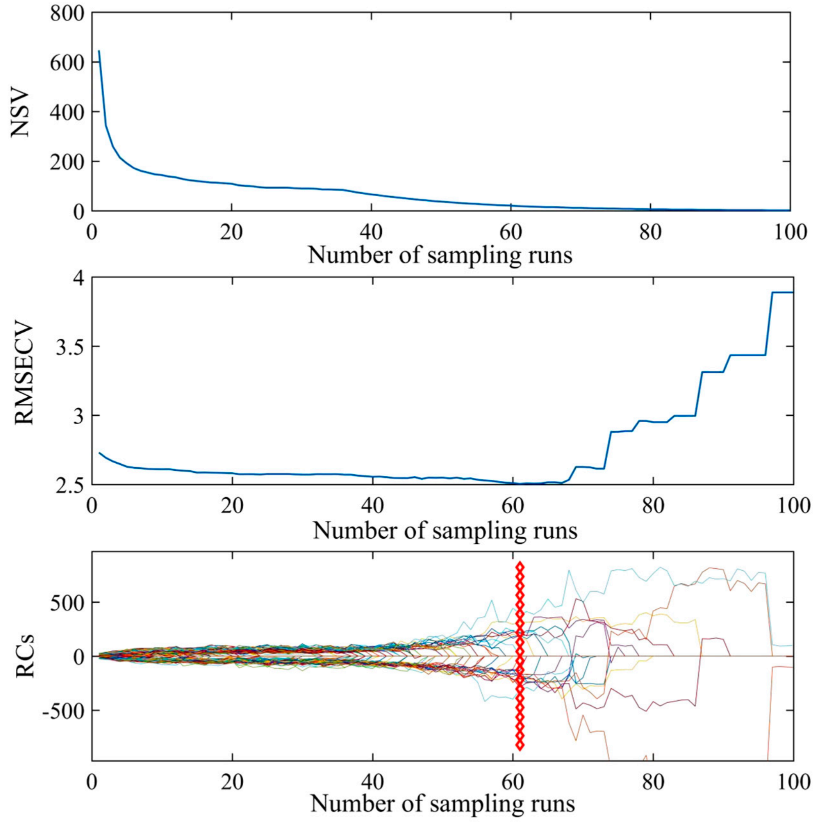

Figure S3. The changes in sample variables (NSV), $RMSE_{cv}$ and regression coefficient paths (RCs) in a subset of CARS arithmetic for Chls.

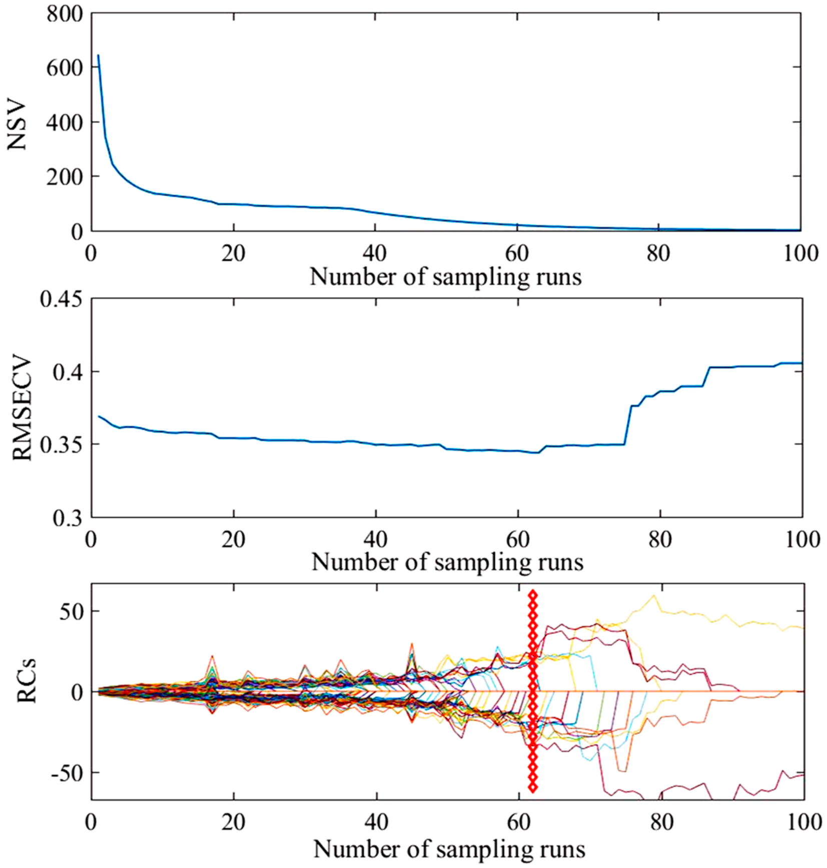

Figure S4. The changes in sample variables (NSV), $RMSE_{cv}$ and regression coefficient paths (RCs) in a subset of CARS arithmetic for Cars.
